# Ribosome inactivation by *Escherichia coli* GTPase RsgA inhibits T4 phage

**DOI:** 10.3389/fmicb.2023.1242163

**Published:** 2023-08-21

**Authors:** Laura Fernández-García, María Tomás, Thomas K. Wood

**Affiliations:** ^1^Department of Chemical Engineering, Pennsylvania State University, University Park, PA, United States; ^2^Microbiology Translational and Multidisciplinary (MicroTM)-Research Institute Biomedical A Coruña (INIBIC) and Microbiology Department of Hospital A Coruña (CHUAC), University of A Coruña (UDC), A Coruña, Spain

**Keywords:** RsgA, phage inhibition, ribosome inactivation, persistence, ribosomes

## Abstract

**Introduction:**

Bacteria must combat phages, and myriad bacterial anti-phage systems have been discovered that reduce host metabolism, for example, by depleting energetic compounds like ATP and NAD^+^. Hence, these systems indirectly inhibit protein production. Surprisingly, direct reduction of ribosome activity has not been demonstrated to thwart phage.

**Methods:**

Here, by producing each of the 4,287 *Escherichia coli* proteins and selecting for anti-phage activity that leads to enhanced growth, we investigated the role of host proteins in phage inhibition.

**Results and discussion:**

We identified that *E. coli* GTPase RsgA inhibits lytic phage T4 by inactivating ribosomes.

## Introduction

The main threat to bacteria are phages, with 20–40% of bacterial mortality a result of phage predation (Hampton et al., 2020). Taking advantage of this and given the dearth of novel antibiotics, interest is increasing in using phages to combat infections (Strathdee et al., 2023). Hence, how a phage redirects the host to synthesize its nucleic acids and its protein capsid during the lytic phase are relevant.

To lyse the host, phages must commandeer both the host transcription and translation machinery; although, little is known about how they control translation. For example, T4 lytic phage lyses the *E. coli* host in 25–30 min and controls host transcription by ADP-ribosylating RNA polymerase at alpha subunit Arg265 (Hinton, 2010), so that by 7 min, 75% of transcription is that of the phage and 75% of the host mRNA is degraded (Wolfram-Schauerte et al., 2022). In contrast, the host proteome remains unchanged during T4 infection while the phage proteome increases after the first min (Wolfram-Schauerte et al., 2022); this shows that protein translation is effectively hijacked by T4 phage. Therefore, during lytic phage attack, host transcription is shut down, host transcripts are degraded, and host ribosomes are re-directed to phage protein synthesis, although the means for controlling host ribosomes is obscure.

Given the ubiquity of phage, cells employ a plethora of phage defenses such as restriction/modification systems [present in 90% of procaryotes (Maguin et al., 2022)], toxin/antitoxin systems (Pecota and Wood, 1996; Song and Wood, 2020a) (present in nearly all bacteria and often in multiple copies), and CRISPR-Cas [present in 40% of bacteria (Maguin et al., 2022)]. Since control of protein production is critical for phages, many anti-phage systems reduce energy compounds in the cell, which inhibits protein translation and phage propagation. For example, NAD^+^ is depleted by the *Bacillus cereus* Thoeris anti-phage system (Leavitt et al., 2022), and ATP is depleted by the *E. coli* RADAR anti-phage system (Duncan-Lowey et al., 2022) and by the GhoT/GhoS toxin/antitoxin system as the toxin GhoT damages the membrane (Cheng et al., 2014). In addition, as the most prevalent phage inhibition system, most toxin/antitoxin systems reduce translation; for example, by degrading mRNA via RNases.

Given that phages require control of ribosomes, we hypothesized that there must be phage inhibition systems that allow the bacterial host to control its protein production machinery or at least deprive phages rapid protein production. By screening all *E. coli* proteins, we discovered that GTPase RsgA inactivates ribosomes to inhibit T4 phage. Moreover, this inhibition of translation results in the cells entering the persister state.

## Results

### Screening all *E. coli* proteins for T4 phage inhibition

**FIGURE 1:**
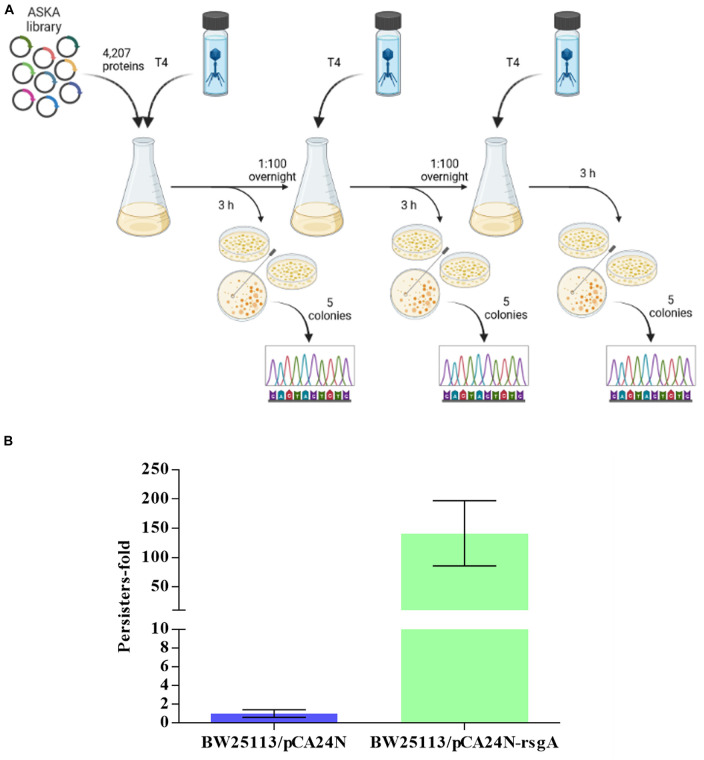
Scheme for screening all 4,287 *E. coli* proteins for T4 phage inhibition **(A)**. Three rounds of selection were used to find cells producing a single protein from pCA24N-based plasmids that inhibit T4 phage propagation (Created with BioRender.com). **(B)** Increase in persister cells with production of RgsA for BW25113/pCA24N-rsgA vs. BW25113/pCA24N.

To identify new anti-phage systems in *E. coli*, we pooled BW25113 cells containing all of the 4,287 ASKA plasmids (Chowdhury et al., 2016) and selected for growth in the presence of T4 phage over three rounds of phage addition (Figure 1). Each ASKA plasmid has a single *E. coli* protein under control of a T5-*lac* promoter. We reasoned that cells that survive T4 attack produce proteins that inhibit phage. From sequencing 25 colonies of cells that survived phage attack, eight different *E. coli* proteins and one intergenic region were identified, including RsgA (Supplementary Table 1). For example, *ygbE* was identified 13 times because this mutation makes the cells mucoid, which blocks T4 phage, so identifying this mutation served as a positive control and shows the robustness of the approach. Also, RsgA is a GTPase whose activity is enhanced 160-fold upon binding 30S ribosome subunits, and since it copurifies with ribosomes at a ratio of 1:200, unlike EF-Tu, EF-G, and IF2, which are found at stoichiometric amounts with ribosomes, it has a non-canonical role in ribosome function (Daigle and Brown, 2004). Its cryo-EM structure indicates RsgA serves as a maturation protein that tests proofreading by the 30S submit before it is released for active translation (Razi et al., 2017). Since only RsgA was related to protein translation, we focused on this protein.

### RsgA inhibits T4 phage

To confirm that RsgA inhibits phage propagation and to confirm the phage inhibition phenotype was not due to a host mutation, we isolated pCA24N-rsgA from *E. coli* AG1 of the ASKA Collection, electroporated pCA24N-rsgA into BW25113, and produced RsgA and found it inhibited T4 phage (MOI 0.01) after a single round of infection for 2 h by 4 ± 3-fold (Supplementary Table 2A). Δ*rsgA* shows slow growth rate in LB Lennox (LB low salt), as well as in LB broth (Baba et al., 2006; Goodall et al., 2018); hence, it is quasi-essential, so experiments with Δ*rsgA* could not be conducted. In addition, plaque-based experiments revealed the efficiency of plating is reduced threefold by producing RsgA (Supplementary Table 2B), and the efficiency of center of infection is reduced 10-fold (Supplementary Table 2C). Therefore, RsgA inhibits T4 phage.

### RsgA inhibits T2 phage

To test whether RsgA inhibits different phage, we produced RsgA from pCA24N-rsgA and found it also inhibited T2 phage (MOI 0.01) after a single round of infection by 200 ± 50-fold (Supplementary Table 2A). Therefore, RsgA is capable of inhibiting several phages.

### RsgA increases persistence to thwart phage attack

Since RsgA is a GTPase related to ribosome function, and persisters cell form upon ribosome dimerization (Kim et al., 2018; Song and Wood, 2020b,c; Song et al., 2021) as a result of energy depletion (Kwan et al., 2013; Cheng et al., 2014), we investigated whether RsgA increases persistence. To assay when persister cells were generated, cell viability with 100 μg/mL ampicillin was determined (i.e., kill curve), and 2.5 h was found to be sufficient to form persister cells (Supplementary Figure 1). Hence, 3 h was used for antibiotic treatment in the persister assays. Upon production of RsgA using pCA24N-rsgA, RsgA increased persister cell formation by 140 ± 60-fold compared to the empty plasmid control (Figure 1B). Note the increase in persistence is not due toxicity of RsgA since the specific growth rate was 1.27 ± 0.06/h for RsgA expressed with 1 mM IPTG for BW25113 pCA24N-*rsgA* vs. 1.35 ± 0.1 for the empty plasmid (BW25113 pCA24N) (Supplementary Figure 2).

**FIGURE 2:**
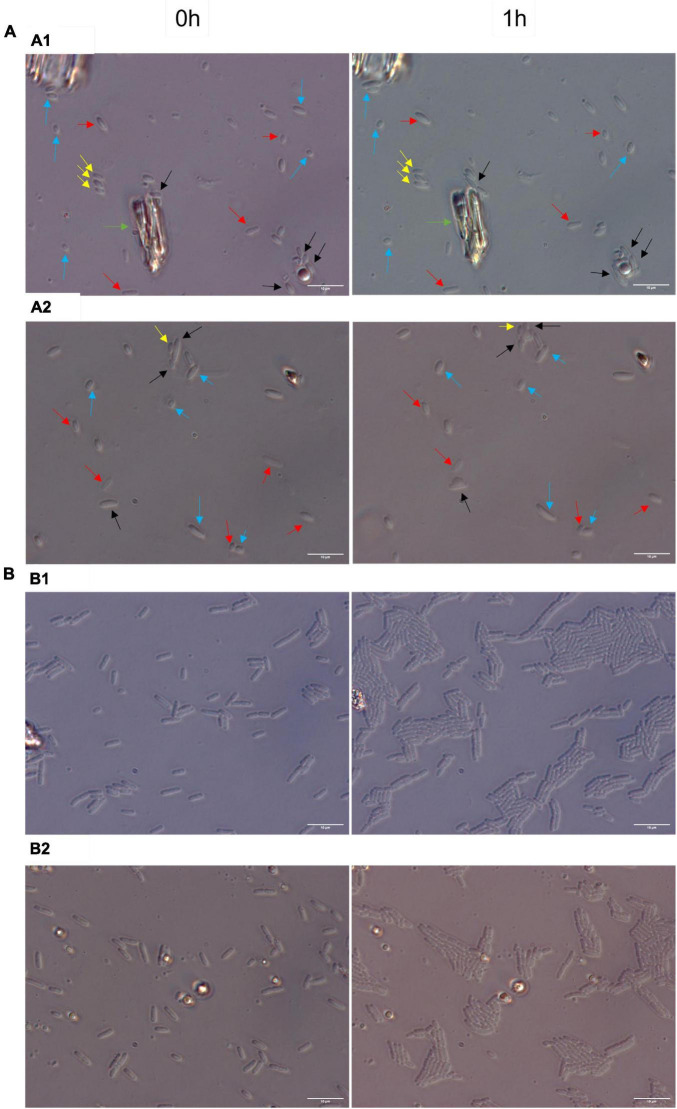
Heterogenous single-cell resuscitation. Representative images (from four independent cultures) of the resuscitation of *E. coli* persister cells with RsgA **(A)** and division of exponential cells **(B)** after 1 h as determined with light microscopy (Zeiss Axio Scope.A1) using LB agarose gel pads (representative images for longer periods; i.e., up to 3 h, are shown in Supplementary Figure 2). The persister cells were generated by overproducing RsgA (1 mM IPTG for 1 h) and treating the cells with 100 μg/ml of ampicillin 3 h. Cells with the empty plasmid (i.e., no RsgA) are not shown due to the cellular debris that stems from almost complete eradication by ampicillin treatment. Black arrows indicate cells with immediate waking (within 1 h), yellow arrows indicate cells with delayed waking (waking between 1–3 h), red arrows indicate cells that wake then die (lyse within 3 h), blue arrows indicate cells that elongate initially, and green arrow indicates mark used to orient images. Data for percentages are shown in Supplementary Table 3.

To explore further whether RsgA induces persistence, we quantified single-cell resuscitation on LB agarose gel pads after producing RsgA using pCA24N-rsgA since heterogeneity in resuscitation indicates persistence (Kim et al., 2018; Goormaghtigh and Van Melderen, 2019; Pu et al., 2019; Windels et al., 2019; Şimşek and Kim, 2019; Fang and Allison, 2023), whereas exponentially-growing cells divide homogeneously without elongation or lags (Kim et al., 2018). After producing RsgA, we found significant heterogeneity in waking with four primary phenotypes including 10 ± 5% waking immediately, 9 ± 3% waking with a lag, 48 ± 5% waking with elongation, and 32 ± 5% dead (Figure 2; Supplementary Figure 3; Supplementary Table 3). In contrast, we found that 97 ± 1% of the sample of exponentially-growing cells divide uniformly without elongation, lags, or death (Figure 2). Hence, RsgA production induces persistence.

## Discussion

**FIGURE 3:**
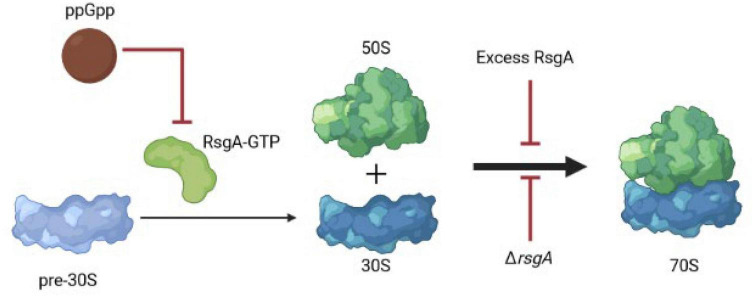
Schematic of RsgA and protein synthesis. Light blue indicates immature ribosomal 30S subunits, which need RsgA-GTP for their maturation, and ppGpp inhibits RsgA GTPase activity as a GTP mimic, thereby reducing translation. Red arrows indicate inhibition. Hence, excess RsgA as well as Δ*rsgA* inhibit protein production.

Our results suggest that along with serving as a checkpoint protein for 30S ribosome subunit maturation (Razi et al., 2017), RsgA also serves to inactivate ribosomes during phage attack. This novel role for RsgA is precedented given it is broadly-conserved and affects growth, ribosomal profiles, peptidoglycan metabolism, cell morphology, and virulence (López-Alonso et al., 2017); hence it plays numerous roles in cell physiology. Critically, we propose here that overproducing RsgA inhibits phage attack by inhibiting formation of active 70S ribosomes and thereby disrupts translation; this slower growth leads to persistence and inhibits phage propagation (Figure 3). Corroborating this model, our data shows producing RsgA reduces growth (Supplementary Figure 3, 1 mM), and Guo et al. (2011) found both purified and overproduced RsgA reduces 70S ribosomes formation in *E. coli.* Note the *rsgA* mutation has also been linked to tetracycline tolerance (Nichols et al., 2011), which fits given the interaction of RsgA and the 30S subunit and the fact that tetracycline inhibits aminoacyl-tRNA binding to the 30S ribosome subunit. Hence, production of RsgA inhibits phage propagation, likely due to its reduction of protein translation. However, a limitation to our results is that they are primarily based on overproducing RsgA compared to the likely physiological production levels.

Since deletion of *rsgA* significantly decreases cell growth in *E. coli* (Himeno et al., 2004), rather than production of RsgA as we found in the current study, it may be more physiologically relevant that inactivating RsgA is used by cells to inhibit phage (Figure 3). For example, in *Staphylococcus aureus*, binding of the alarmone guanosine tetraphosphate (ppGpp produced by RelA and SpoT in *E. coli*) to RsgA to inactivate its GTPase activity, slows growth and increases antibiotic tolerance (Corrigan et al., 2016). Therefore, both overproduction and inactivation of RsgA slow cell growth. Hence, we propose that ppGpp could signal the stress of phage attack, which then binds and inactivates RsgA, leading to a reduction in protein production and phage inhibition; the role of ppGpp as a signal for phage attack remains to be investigated. However, it is well-established that slow growth inhibits phage propagation (You et al., 2002) since it deprives phages of nucleotides and proteins, and ppGpp is considered the stress signal that leads to persistence through ribosome inactivation (Song and Wood, 2020c; Wood and Song, 2020). Moreover, *E. coli* was found to “hibernate” as a result of the RpoS-mediated stress response during T4 infection of stationary cells (Bryan et al., 2016).

Our results inhibiting both T2 and T4 phage by activating RsgA suggest that reducing translation may be a general means to inhibit phage. Moreover, our results also suggest that the formation of dormant cells; i.e., persisters, should inhibit phage production. Corroborating this, recently, we found that activation of the MqsR/MqsA/MqsC tripartite toxin/antitoxin system in *E. coli* by phage T2 attack results in cells entering the persister state, which enables the EcoK McrBC restriction modification system to eliminate T2 phage (Fernández-García et al., 2023). In a similar fashion, the *Listeria* spp. type VI CRISPR-Cas system induces dormancy, which allows, restriction/modification systems to eliminate phage (Williams et al., 2023). Therefore, dormancy/persistence appears to be a general approach for cells to thwart phages.

## Materials and methods

### Bacteria and growth conditions

Bacteria and plasmids are shown in Supplementary Table 4, and cells were cultured at 37°C in lytic broth (Maniatis et al., 1982) (LB) supplemented with 30 μg/ml of chloramphenicol (LB-Cm30) to retain plasmids. The pCA24N-rsgA plasmid was confirmed by sequencing at Plasmidsaurus.

### Identification of T4 phage inhibition systems

BW25113 cells were electroporated with 40 ng of the pooled ASKA (GFP^–^) library (Chowdhury et al., 2016), incubated for 1 h in LB to recover, diluted, and grown to a turbidity of ∼ 0.5 at 600 nm (∼8 h) in LB-Cm30. For the first round, T4 phage (1.12 × 10^9^ pfu/ml) was added, and the cultures were incubated for 3 h with 250 rpm shaking, then plated on LB-Cm30. For the second round and third rounds, cultures were washed with phosphate buffered saline (PBS, 8 g of NaCl, 0.2 g of KCl, 1.15 g of Na_2_HPO_4_, and 0.2 g of KH_2_PO_4_ in 1,000 mL of dH_2_O) and diluted by adding 150 μL into 15 ml of fresh LB-Cm30 and incubated overnight and 250 rpm. Overnight cultures were diluted 1:100 into fresh LB-Cm30 and incubated with 250 rpm shaking to a turbidity of 0.5. Cells were transferred (15 ml) to a new flask, 500 μL of T4 phage (1.12 × 10^9^ pfu/ml) was added, and the cultures are incubated for 3 h at 250 rpm, then plated on LB-Cm30. The pCA24N plasmids from eight colonies were sequenced with pCA24N-specific primers to identify the insert (Forward: 5′-TGACCATGATTACGGATTCACTGGCC-3′ and Reverse: 5′- ACAGACAAGCTGTGACCGTCTCCGG-3′) by making PCR products, which were sequenced by Quintara Biosciences, Inc. The genes were identified using a BLAST search against the BW25113 genome sequence.

### Phage resistance assays

To investigate survival, single colonies were cultured overnight in LB-Cm30, then diluted 100X and grown to a turbidity of 0.1 at 600 nm. IPTG (1 mM) was added, cells were incubated for an hour, T2 or T4 phage was added (multiplicity of infection ∼ 0.01) for 2 h, then the cells were washed twice with PBS and enumerated on LB-Cm plates to measure survival.

The efficiency of plating assay (Kutter, 2009) was performed with BW25113 harboring pCA24N or pCA24N-rsgA using 10^–1^ to 10^–7^ serial dilutions of phage T4 and double agar plates inoculated with 100 μL of overnight cultures and dried for a minimum 15 min. Diluted phage was added to soft agar plates (5 μL drops), and after overnight incubation, the number of plaque forming units (PFU)/mL was determined. The efficiency of the center of infection assay (Sing and Klaenhammer, 1990) (i.e., a pre-adsorbed productive infection assay) was used to determine the ability of T4 phage to infect BW25113 harboring pCA24N or pCA24N-rsgA; overnight bacterial cultures were diluted 1:100 into 15 mL of fresh buffered LB Cm30 and were incubated to a turbidity at 600 nm of ∼0.5, then T4 phage (MOI ∼0.1) was added to the cultures and incubated for 8 min (adsorption time). After washing twice with PBS (centrifuging at 5,000 rpm for 10 min) to remove free phages, the cultures were diluted into phage buffer (dilutions 1–5), and 5 μL drops were added to soft agar plates containing BW25113. Plates were placed at 37°C for overnight incubation and PFU/mL determined.

### Persister assay

Single colonies were cultured overnight in LB-Cm30, then diluted 100X and grown to a turbidity of 0.1 at 600 nm. IPTG (1 mM) was added, and the cells were incubated for an hour, then 5 mL of cells were harvested by centrifugation at 5,000 rpm for 10 min and resuspended in 5 ml of LB containing 100 μg/mL of ampicillin (10 MIC). The cultures were incubated for 3 h with shaking at 250 rpm in a 15 mL culture tube, washed twice with PBS, then 100 μL were serially diluted with PBS to determine the number of viable cells. The kill curve assay to verify persister cells were formed during ampicillin treatment was performed by treating late exponential cells (turbidity at 600 nm of ∼0.8) with 100 μg/ml ampicillin for 4 h with shaking at 250 rpm in a 15 mL flask. Samples were taken every 30 min, washed with PBS, and 100 μL was serially diluted with PBS to determine the number of viable cells.

### Persister resuscitation assay

Single-cell microscopy of resuscitating persister cells was performed as described previously (Kim et al., 2018) using LB agarose gel pads that were observed up to 3 h via a light microscope (Zeiss Axioscope.A1, bl_ph channel at 1,000 ms exposure). The persister cells were generated by adding 1 mM IPTG for one and a half hours and ampicillin (100 μg/mL) was added for 3 h. The microscope was maintained at 37°C by placing it in a vinyl glove box (Coy Labs, Grass Lake, MI, USA), which was warmed by an anaerobic chamber heater (Coy Labs, 8535-025).

## Data availability statement

The original contributions presented in this study are included in the article/Supplementary material, further inquiries can be directed to the corresponding author.

## Author contributions

LF-G conducted the experiments and helped to write the manuscript. MT helped to edit the manuscript. TW conceived the idea and helped to write the manuscript. All authors contributed to the article and approved the submitted version.
