# Supplementary material for: Ribosome inactivation by Escherichia coli GTPase RsgA inhibits T4 phage
Source: Front Microbiol. 2023 Aug 21;14:1242163. doi: 10.3389/fmicb.2023.1242163 (PMC10475562; doi:10.3389/fmicb.2023.1242163)

Supporting Information

Ribosome Inactivation by *Escherichia coli* GTPase RsgA Inhibits T4 Phage

Laura Fernández-García[1,2], María Tomás[2], and Thomas K. Wood[1*]

[1]Department of Chemical Engineering, Pennsylvania State University,
University Park, Pennsylvania, 16802-4400, USA

[2]Microbiology Translational and Multidisciplinary (MicroTM)-Research Institute Biomedical A Coruña (INIBIC) and Microbiology Department of Hospital A Coruña (CHUAC); University of A Coruña (UDC), A Coruña, Spain

*For correspondence. E-mail tuw14@psu.edu

Tel. (+)1 814-863-4811; Fax (1) 814-865-7846

Table S1. *E. coli* genes identified via the pooled ASKA screen for inhibiting T4 phage.

Gene	Times identified	Function
ygbE	13	Inner membrane protein
ulaA	1	Membrane component of the ascorbate PTS system
frvB	1	Similar to fructose PTS proteins
csiE	1	Stationary phase inducible protein
ycbX	2	Molybdenum cofactor-dependent pathway protein
eutS	1	Protein from the *eut* operon
mcrB	1	DNA binding protein
rsgA	1	GTPase associated with 30S ribosomes
fabY-yiiE	2	Intergenic region

Table S2. Inhibition of T4 and T2 phage by RsgA. Survival results **(A)** after BW25113 with the indicated plasmid was induced with 0.1 mM IPTG at turbidity 0.1 (600 nm) for 1 h and infected with T4 and T2 phage (MOI 0.01) for 2 h, then washed three times with PBS before plating (to remove phage). Results are the average of three independent cultures. Efficiency of plating results **(B)** were conducted using overnight cultures of BW25113 with the indicated plasmid along with 10^{-1} to 10^{-7} phage dilutions to determine pfu/mL. Results are the average of two independent cultures. Efficiency of center of infection results **(C)** were conducted using BW25113 with the indicated plasmid grown to turbidity 0.5 (600 nm) and infected with T4 phage (MOI 0.1) for 8 min (adsorption time), then washed twice with PBS before plating (to remove free phages) and determining pfu/mL. Results are the average of two independent cultures.

A

**Phage**	**Strain**	**CFU/mL before phage**	**CFU/mL after phage**	**Survival (%)**	**Std Deviation (%)**
T4	pCA24N	1×10^{8}	6.8×10^{2}	0.0007	0.0004
	pCA24N-rsgA	7.4×10^{7}	1.9×10^{3}	0.0026	0.001
T2	pCA24N	1×10^{8}	1.2×10^{2}	0.0001	0.00003
	pCA24N-rsgA	7.4×10^{7}	1.8×10^{4}	0.024	0.003

B

**Phage**	**Strain**	**Average PFU/mL**	**EOP vs host**
T4	pCA24N	$5.4 \pm 0.9 \times 10^{10}$	1
	pCA24N-rsgA	$1.8 \pm 0.5 \times 10^{10}$	0.33

C

**Phage**	**Strain**	**Average PFU/mL**	**ECOI vs host**
T4	pCA24N	$6.6 \pm 0.9 \times 10^{8}$	1
	pCA24N-rsgA	$7.0 \pm 1 \times 10^{7}$	0.10

Table S3. ***E. coli*** **persister cell resuscitation for BW25113/pCA24N-rsgA after treatment with ampicillin.** Values for the number of cells with the indicated phenotype are listed parenthetically. Resuscitation is for *E. coli* persister cells with RsgA after 0 to 3 h as determined with light microscopy (Zeiss Axio Scope.A1) using LB agarose gel pads. The persister cells were generated by producing RsgA (1 mM IPTG for 1.5 h) and by treating the cells with 100 µg/ml of ampicillin for 3 hours.

Sample	No of cells	% of waking in 1 h	% of waking in 1 to 3 h	% Elongated cells	% of dead
1	68	6% (4/68)	19% (13/68)	44% (30/68)	31% (21/68)
2	22	14% (3/22)	5% (1/22)	41% (9/22)	41 % (9/22)
3	26	4% (1/26)	15% (4/26)	50% (13/26)	31 % (8/26)
4	20	15% (3/20)	5% (1/20)	55% (11/20)	25% (5/20)
**Average**		**10 ± 5%**	**9 ± 3%**	**48 ± 5%**	**32 ± 5%**

Table S4. *E. coli* bacterial strains and plasmids utilized. Cm^R is chloramphenicol resistance.

Strains and Plasmids	Features	Source
**Strains**		
BW25113	*rrnB3 ΔlacZ4787 hsdR514 Δ(araBAD)567 Δ(rhaBAD)568 rph-1*	(Baba et al., 2006)
AG1/pCA24N-rsgA	*recA1 endA1 gyrA96 thi-1 hsdR17 (r K− m K+) supE44 relA1*	(Kitagawa et al., 2005)
**Plasmids**		
pooled ASKA library	pCA24N-based, 4,287 plasmids, Cm^R, *lacI*^{q}, $P_{T5\text{-}lac}$::gene	(Kitagawa et al., 2005), (Chowdhury et al., 2016)
pCA24N-rsgA	pCA24N-based, Cm^R, *lacI*^{q}, $P_{T5\text{-}lac}$::*rsgA*	(Kitagawa et al., 2005)

Supplemental Fig. S1. Kill curve after ampicillin treatment showing persister cells form after 2.5 hr. BW25113/pCA24N cells were grown at 37°C in LB medium until a turbidity at 600 nm of approximately 0.8, then 100 µg/mL of ampicillin was added. Cell viability was monitored every 30 min by determining the colony forming units (CFU). Since persisters were clearly formed after 2.5 h, 3 h ampicillin treatments were used. Standard deviations are shown.

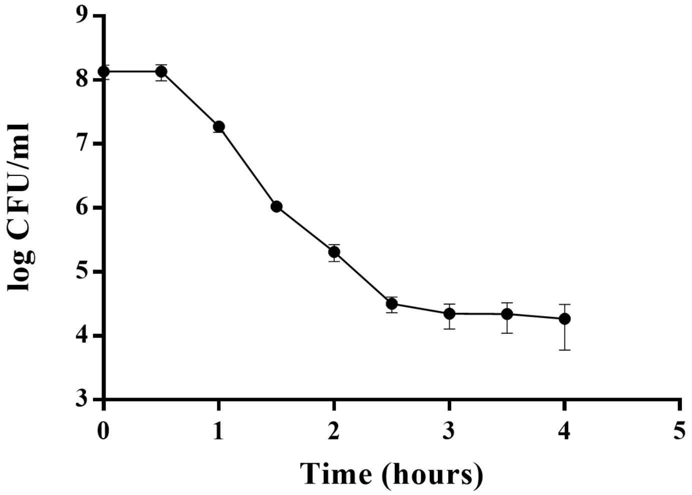

Supplemental Fig. S2. Growth during production of RsgA. Cells were grown at 37°C in LB with induction of *rsgA* from plasmid pCA24N in host BW25113 using 1 mM IPTG (blue line, BW25113/pCA24N-rsgA; green line, BW25113/pCA24N) or without induction (red line, BW25113/pCA24N-rsgA; orange line, BW25113/pCA24N).

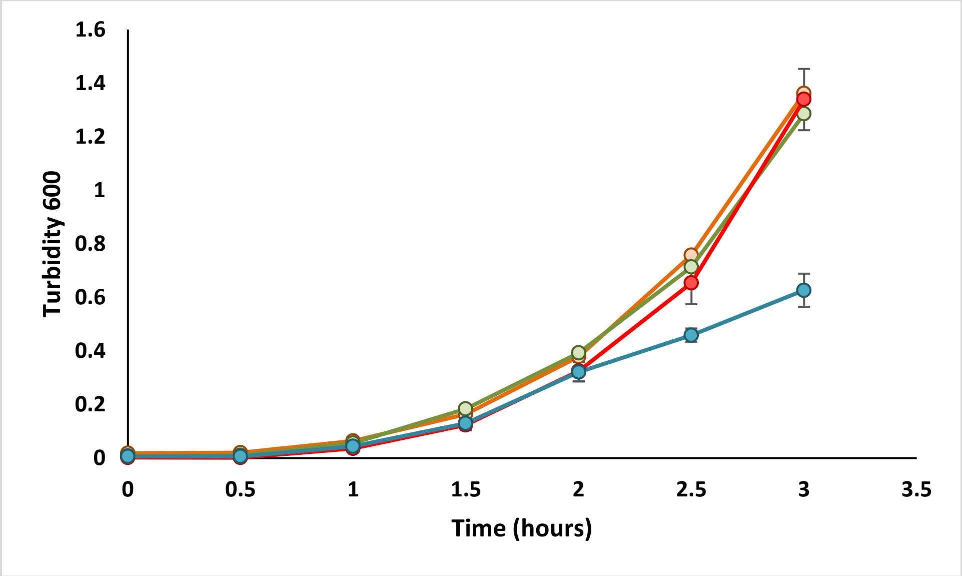

Supplemental Figure S3. Long-term heterogenous single-cell resuscitation showing microcolony formation. Representative images (from five independent cultures) of the resuscitation of *E. coli* persister cells with RsgA after 0 to 3 h as determined with light microscopy (Zeiss Axio Scope.A1) using LB agarose gel pads. The persister cells were generated by producing RsgA (1 mM IPTG for 1 hour) and by treating the cells with 100 µg/ml of ampicillin for 3 hours. Cells with the empty plasmid (i.e., no RsgA) are not shown due to the cellular debris that stems from almost complete eradication by ampicillin treatment. Black arrows indicate cells with immediate waking (within 1 h), yellow arrows indicate cells with delayed waking (waking between 1 – 3 h), red arrows indicate cells that wake then die (lyse within 3 h), blue arrows indicate cells that elongate, and green arrows indicate marks used to orient images. Data for percentages from 0 to 1 h are shown in **Table S3**.

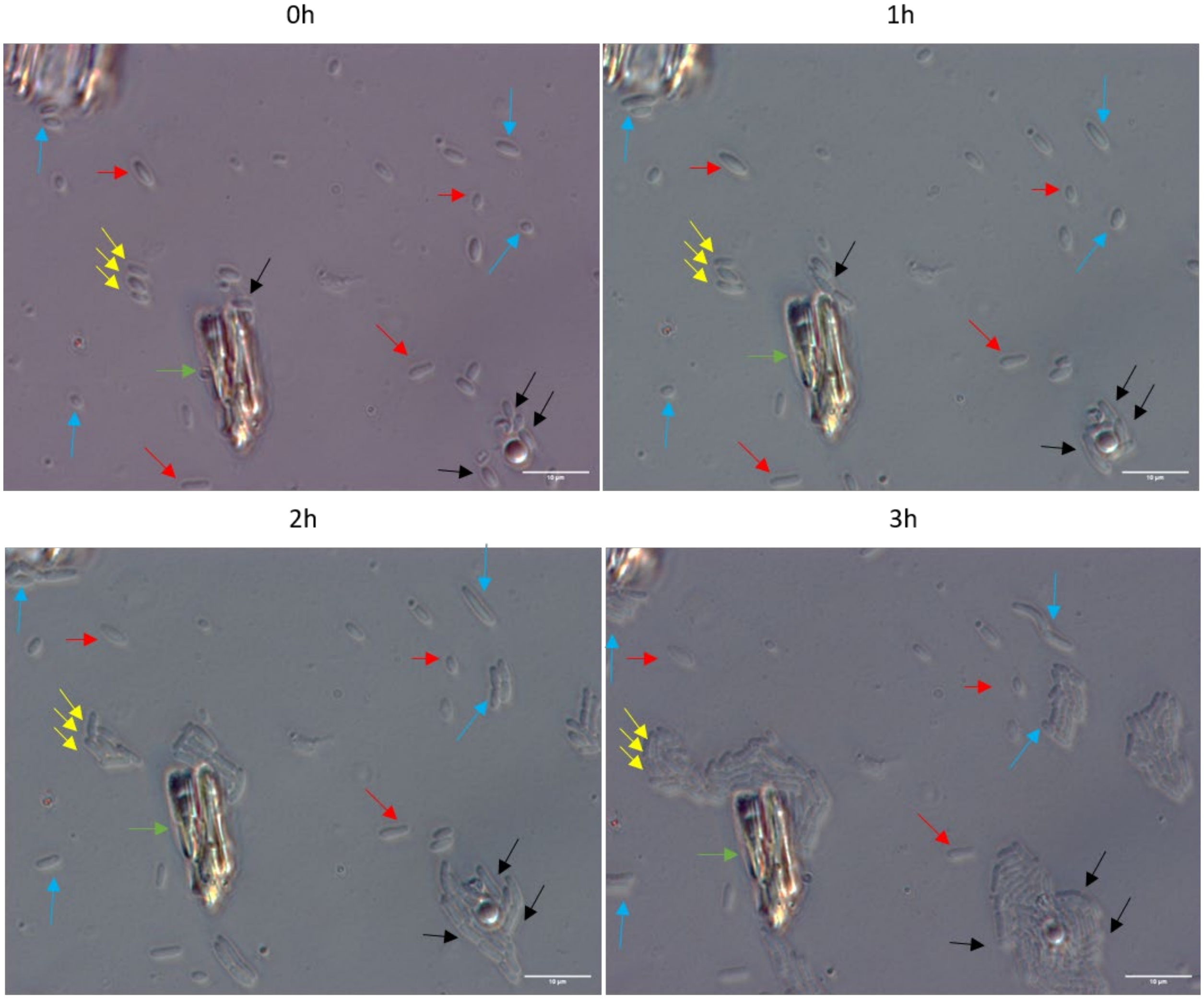

Supplement: Supplementary file 1 [file Data_Sheet_1.pdf]
